# Physicochemical Profiling of Baicalin Along with the Development and Characterization of Cyclodextrin Inclusion Complexes

**DOI:** 10.1208/s12249-019-1525-6

**Published:** 2019-09-16

**Authors:** Géza Jakab, Dóra Bogdán, Károly Mazák, Ruth Deme, Zoltán Mucsi, István M. Mándity, Béla Noszál, Nikolett Kállai-Szabó, István Antal

**Affiliations:** 1 Department of Pharmaceutics, Semmelweis University, Hőgyes E. Street 7-9, Budapest, 1092 Hungary; 2 Department of Organic Chemistry, Semmelweis University, Hőgyes E. Street 7-9, Budapest, 1092 Hungary; 3 MTA TTK Lendület Artificial Transporter Research Group, Institute of Materials and Environmental Chemistry, Research Center for Natural Sciences, Hungarian Academy of Sciences, Magyar Tudósok krt. 2., Budapest, 1117 Hungary; 4 Department of Pharmaceutical Chemistry, Semmelweis University, Hőgyes E. Street 7-9, Budapest, 1092 Hungary; 5 Femtonics Ltd., Tűzoltó Street 59, Budapest, 1094 Hungary

**Keywords:** baicalin, physicochemical analysis, computational modelling, cyclodextrins, solubility enhancement

## Abstract

Baicalin is a flavone glycoside extracted from *Scutellaria baicalensis*, a traditional Chinese herbal medicine. Numerous pharmacological effects of baicalin were reported (*e.g.* antioxidant, anxiolytic); nevertheless, the most important physicochemical properties influencing the pharmacokinetic behaviour and the concomitant oral bioavailability have not yet been described in a comprehensive study. The aim of this project was to characterize the acid-base, lipophilicity, biorelevant solubility and permeability properties of the drug substance and providing scientific data to support the dosage form design. Another important objective was the comparative evaluation of six various baicalin-cyclodextrin (CD) inclusion complexes along with the creation of a suitable Drug Delivery System (DDS) for this BCS IV drug. Biorelevant profiling was carried out by NMR-pH titrations, saturation shake-flask and distribution coefficients (log*P*) measurements, while CD inclusion studies were fulfilled by experimental methods (phase solubility, ^1^H/^13^C NMR, 2D ROESY) and computational approaches. Due to low aqueous solubility (67.03 ± 1.60 μg/ml) and low permeability (*P*_app_ = 0.037 × 10^−6^ cm/s), baicalin is classified as BCS IV. The γ-CD complexation significantly increased the solubility of baicalin (~ 5 times). The most promoted chemical shift change occurred in baicalin-γ-CD complex. Computational studies showed disparate binding pattern for baicalin in case of β- and γ-CD; furthermore, the calculated complexation energy was − 162.4 kJ mol^−1^ for β-CD, while it was significantly stronger for γ-CD (− 181.5 kJ mol^−1^). The physicochemical and structural information of baicalin and its CD complexes introduced herein can create molecular basis for a promising DDS with enhanced bioavailability containing a bioactive phytopharmacon.

## INTRODUCTION

*Scutellaria baicalensis* Georgi (*S. baicalensis* G.) is one of the most elemental herbs in traditional Chinese herbal medicine known as Huang Qin [1]. The root is officially listed in the Chinese Pharmacopoeia and was assumed in European Pharmacopoeia (Ph.Eur.) 9th Edition last year [2]. The major bioactive components in the dried root are baicalin (7-D-glucuronic acid-5,6-dihydroxyflavone) and its aglycone, baicalein (5,6,7-trihydroxyflavone) [3]. Baicalin has attracted increasing scientific attention because of its various pharmacological activities such as antioxidant, antitumor, anti-inflammatory and hepatoprotective effects [4, 5].

Baicalin is reported to have low water solubility (0.054 mg/ml) and low permeability (*P*_app_ = 0.037 × 10^−6^ cm/s) [6]. The absolute bioavailability of baicalin is as low as 2.2 ± 0.2% [7]. An integrated knowledge of biorelevant physical and chemical parameters of the active substance is of importance to select the proper formulation and delivery method of the drug [8]. Besides of passive transport, carrier-mediated processes play a prominent role in the intestinal absorption of baicalin. Different ATP-binding cassette (ABC) transporters were identified, which are responsible for the enterohepatic recirculation and absorption mechanisms of baicalin [9].

Baicalin has three acidic functional moieties, one carboxyl and two phenolic hydroxyl groups. Proton transfer processes can be regarded either from the aspect of dissociation or association. Because of the acidic nature of baicalin, these processes will be characterized here in terms of acid dissociation constants (*K*_a_). Carboxylic acids are typically orders of magnitude stronger than phenols. The determination of baicalin dissociation constants is hampered by its poor water solubility and sensitivity of oxidation of the catechol structure on its A ring [10]. The acid/base properties of baicalin have been investigated earlier, resulting in p*K*_a1_, p*K*_a2_ and p*K*_a3_ values of 5.05, 7.6 and 10.1, respectively [10]. However, our NMR investigations revealed that the molecule rapidly decomposes above pH 9, making the reported values in alkaline solutions highly dubious. We decided to undertake the characterization of baicalin by NMR-pH titrations, where the molecule was protected from oxidation by the antioxidant ascorbic acid up to pH ≈ 13.

A thorough physicochemical characterization necessitates the determination of the octanol/water partition coefficient (log *P*) too, which is in close correlation with membrane-penetration capability and is the most frequently determined lipophilicity descriptor. While there have been attempts in characterizing the lipophilicity of baicalin [10], we decided to carry out a more detailed study in this area as well.

Aqueous solubility is a fundamental attribute of an active pharmaceutical ingredient (API) and its examination is a mandatory step during the drug discovery process [11]. New drug molecules recently tend to have larger molecular weights, resulting often in decreased solubility. Bloomer et al. reveal how the solubility of the API varies as the pH and level of surfactants varies in the gastrointestinal tract in the fasted and fed state [12]. Furthermore, drug-food interactions can be detected and investigated by using biorelevant media (BRM) [13]. Simulated intestinal fluid in fasted state (FaSSIF), in fed state (FeSSIF) and gastric fluid in fasted state (FaSSGF) were introduced first. Simulated fluids include different components (*e.g.* bile salts, lecithin, pepsin, ions) at given ratio, which adequately predict the *in vivo* behaviour of drug formulations [14].

Self-nanoemulsifying systems, nanocrystals and cyclodextrin complexes were developed to counterbalance the low solubility and concomitant low oral bioavailability of baicalin [15–17]. Cyclodextrins (CDs) are cyclic oligosaccharides composed of α-1,4-linked D-glucopyranose units possessing a hydrophilic exterior and hydrophobic cavity, where lipophilic molecules can form a non-covalently bonded inclusion complex [18]. The cyclodextrin complexation of baicalin is proved to be a promising method to increase its solubility; however, only a limited number of studies were reported, in which hydroxypropyl-*β*-, and *β*-CD were used [16, 19]. Baicalin is a class IV drug, so the enhancement of permeability is also an essential part from bioavailability point of view. Cyclodextrin derivatives can be hydrophilic or relatively lipophilic based on their substitution and these properties can give insight into their ability to act as permeability enhancers [20]. Furthermore, it was described that CDs can improve bioavailability by inhibiting P-gp (subfamily of ABC transporters) induced efflux mechanisms [21]. The efflux of baicalin is likely mediated by these kinds of ABC transporters [9]. This fact verifies the relevance of preparation, characterization and analysis of different baicalin-CD complexes.

The objective of this study was to quantify the most important biorelevant physicochemical properties of baicalin in terms of acid/base, lipophilicity and thermodynamic solubility in different compendial and physiological media. To counterbalance the low solubility and permeability of this potent phytopharmacon, the preparation and comparative evaluation of various baicalin-CD inclusion complexes were put into the focus. In order to reveal the molecular interactions inside the cavity and to characterize the 3D geometry of the complex, each species was subjected to phase solubility, ^1^H NMR and 2D ROESY experiments along with molecular modelling of the binding pattern into cyclodextrins. Comparison of computational study and experimental results were also in our interest.

## MATERIALS AND METHODS

### Materials

Baicalin (Batch: BA-161118) and its methyl ester were purchased from Actin Chemicals, Inc. (Chengdu, China). All CDs (*α*-CD, *β-*CD, *γ*-CD, (2-hydroxypropyl)-*β*-CD (*HP-β*-CD), random methylated *β*-CD (*RAMEB*-CD) and sulfobutylether-*β*-CD (*SBE*-*β*-CD)) were kindly donated by Cyclolab R&D Ltd. (Budapest, Hungary). Sodium taurocholate, lecithin, pepsin, indicator molecules for the NMR-pH titrations (TRIS, acetamidine hydrochloride and 1-methylguanidine hydrochloride) were purchased from Sigma-Aldrich (Budapest, Hungary), the deuterated solvent D_2_O from VWR International (Debrecen, Hungary) and the DMSO-*d*_*6*_ from Euriso-top (Saint-Aubin, France). Other chemicals of analytical grade were obtained from commercial suppliers and used without further purification. Bidistilled water was used for all NMR solutions; for solubility analysis, freshly prepared distilled water was applied.

### Preparation of Compendial and Biorelevant Media

The pH value of each solution was controlled with a Hanna® pH 210 microprocessor pH meter (Hanna Instruments, USA) and media were prepared according to literature at room temperature [22].

A total of 0.94 g of 1 M HCl and 0.35 g of NaCl were diluted and dissolved to 1000 ml with distilled water and used as simulated gastric fluid (SGF) with pH 1.2. This medium served as solvent for the measurement of intrinsic solubility of baicalin.

A total of 250 ml of 0.2 M K_2_HPO_4_ and 77 ml of 0.2 M NaOH were homogenized and diluted to 1000 ml with distilled water. This medium served as simulated intestinal fluid (SIF) with pH 6.8.

Blank buffer of FaSSGF pH 1.6 (1.99 g NaCl dissolved in 900 ml distilled water, adjusted the pH to 1.6 with 1 M HCl and filled to 1000 ml with distilled water) and phosphate buffer pH 6.5 (0.42 g NaOH pellets + 3.95 g NaH_2_PO_4_ monohydrate + 6.19 g NaCl dissolved in 900 ml distilled water, adjusted the pH to 6.5 with either 1 M NaOH or 1 M HCl and filled to 1000 ml with distilled water) were prepared as stock solutions.

FaSSGF was produced from blank buffer of FaSSGF pH 1.6, which was completed by 0.08 mM sodium taurocholate, 0.02 mM lecithin, 0.1 mg/ml pepsin and has osmolality of 120 ± 2.5 mOsmol/kg. To obtain the FaSSIF medium, 100 ml of blank phosphate buffer (pH 6.5) was made up by 3 mM sodium taurocholate and 0.75 mM lecithin. FaSSIF has an osmolality of 270 ± 10 mOsmol/kg.

### Determination of Thermodynamic Solubility by Saturation Shake-Flask Method

The thermodynamic solubility of baicalin in various media was determined by saturation shake-flask method based on the method of Baka et al. [23]. Briefly, 10 mg of baicalin was added to 5 ml of each solvent in sealed vials. All samples were stirred (approx. 500 rpm) and thermostated (IKA RT-5 power heatable magnetic stirrer, IKA Work Inc., USA) at 37 ± 0.5°C for 24 h allowing it to achieve thermodynamic equilibrium. After this period, 24 h of sedimentation cycle was adopted without stirring at 37 ± 0.5°C. To improve the efficacy of phase-separation, the saturated supernatant of samples was centrifuged at 14,000 rpm for 15 min (Herolab MicroGen 16, Herolab GmbH, Wiesloch, Germany). The aliquots (250 μl) taken for solubility experiments were suitably diluted with blank buffers and the absorbance was measured at λ = 316 nm by UV spectroscopy (Agilent 8453 UV-Visible Spectrophotometer, Agilent Technologies Ltd., USA). The drug exhibited absorption maxima (λ_max_) at 214, 279 and 316 nm in distilled water. Ideally, λ_max_ selected for the analysis of drug should not show any interference due to solvents and excipients present in the dissolution medium. At 279 nm, baicalin showed higher molar absorptivity; however, this wavelength was not selected for the quantification of the drug because there was interference of excipients in biorelevant media at this wavelength. In the solubility study, thus, λ_max_ 316 nm was selected for the quantification of baicalin. In case of the distribution study, the above mentioned obstacle did not appear, so λ_max_ 279 nm could be easily used. All experiments were repeated three times and results were calculated from the linear calibration curve of baicalin in each media. Data are expressed as mean ± SD (μg/ml).

### NMR-pH Titrations

NMR-pH titrations were performed on a Varian VNMRS spectrometer (600 MHz for ^1^H). Spectra were recorded at 25°C. Titrations were carried out in solutions containing 95% (*v*/*v*) H_2_O and 5% (*v*/*v*) D_2_O, with the addition of small amounts of 0.1 M HCl and NaOH. One molar NaOH and solid NaOH crystals were also used to achieve highly basic pH values. The ionic strength was kept at 0.15 M by the presence of NaCl. The concentration of baicalin and its methyl ester was 1.0 × 10^−3^ M and ascorbic acid was used in large excess (5.0 × 10^−3^ M) to prevent the oxidation of the studied catechol molecules in alkaline solutions. The sample volume was 600 μl. NMR spectra were referenced to the internal standard DSS (sodium 3-(trimethylsilyl)-1-propanesulfonate). The water signal was suppressed by presaturation. Spectra were processed with VNMRj 3.2a software. pH values were read on a Metrohm 2.780.0010 precision pH meter with a 6.0258.600 Unitrode glass Pt 1000 electrode (Metrohm AG, Herisau, Switzerland). The pH-potentiometric system was calibrated using pH 1.68, 4.01, 6.87, 9.18 aqueous buffer solutions. In highly basic solutions, pH was measured by NMR-pH indicator molecules as well to avoid the uncertainty of the glass electrode pH-meter readings in such solutions [24]. The concentration of these pH indicators was 1.0 mM. For the analysis of NMR titration curves of proton chemical shifts *versus* pH, the software Origin Pro 8 (OriginLab Corp., Northampton,MA, USA) was used.

### Distribution Coefficient Measurements by the Stir-Flask Method

The distribution coefficients were calculated from the absorbance of the molecules before and after partitioning at several octanol/water phase ratios [25]. For the pH control, a pH 7.40 phosphate buffer and a standardized HCl solution were used, with an ionic strength of 0.15 M in both cases. The pH of the phosphate buffer was measured using a Metrohm 6.0204.100 combined pH glass electrode and a Metrohm 780 pH meter. The concentration change of baicalin was followed by UV-absorbance measurements. Because of the poor water solubility in acidic solutions, the absorbance of the octanol phase (with an initial baicalin concentration of 4.2 × 10^−5^ M) had to be monitored in the partition experiments with 0.15 M HCl. However, at pH 7.40, the solubility in water was high enough to follow the concentration change of baicalin in the aqueous phase, starting from the initial concentration of 5.6 × 10^−5^ M. Several water/octanol phase ratios were used, depending on the pH and the expected log *D* value, to ensure that the absorbance of baicalin after partitioning should become approximately half as much as the original value before partitioning. Then, the two phases were intensively stirred for 2 h in thermostated double-walled glass cells at constant temperature (25 ± 0.1°C). After separation of the equilibrated phases and centrifugation, the concentration of baicalin was determined by UV spectrophotometry (Perkin-Elmer Lambda 15) at several values at 279 nm (λ_max_). The distribution coefficients were calculated from the absorbance of baicalin before and after partitioning.

### Phase Solubility Studies

The phase solubility studies were carried out according to the method of Higuchi and Connors [26]. Experimentally, an excess amount of baicalin (10 mg**,** 2.25 mM) was added to increasing concentrations (5, 10, 20, 40, 80 mM) of various distilled water-based CD stock solutions at pH 4.5 (in case of *β*-CD—because of its low aqueous solubility—1.25, 2.5, 5, 7.5, 10 mM stock solutions were prepared). The vials were agitated by ultrasonication (Branson 5200, Danbury, USA) for 4 h at constant room temperature (25°C) followed by 72 h of equilibration phase (25°C). After sedimentation, the saturated supernatant was taken and centrifuged at 14,000 rpm for 15 min with Herolab MicroCen 16 centrifuge (Herolab GmbH, Wiesloch, Germany). Samples were suitably diluted with distilled water and drug concentration was obtained *via* UV spectroscopy at wavelength 278 nm (Agilent 8453 UV-Visible Spectrophotometer, Agilent Technologies Ltd., USA). All experiments were repeated three times and results were calculated from the linear calibration of baicalin in distilled water (*R*^2^ = 0.9992). Assuming 1:1 complex stoichiometry, the stability constants (*K*_*1:1*_) of baicalin-CD inclusion complexes were calculated based on phase solubility diagrams according to Higuchi-Connors equation:

1 $$ {K}_{1:1}=\frac{slope}{interception\ast \left(1- slope\right)} $$

### Signal Assignment of Baicalin and Characterization of Baicalin-Cyclodextrin Inclusion Complexes Using ^1^H NMR and 2D ROESY Experiments

Assignment and CD complexation NMR experiments were recorded at 25°C on a Varian Mercury Plus spectrometer at 400 MHz. For complete signal assignment, 15 mg of baicalin was dissolved in 0.6 ml DMSO-*d*_*6*_ and transferred to 5 mm NMR tube. For the investigation of inclusion complexes, cyclodextrin was dissolved in 0.7 ml D_2_O and baicalin was dissolved in 0.3 ml DMSO-*d*_*6*_; these two stock solutions were mixed and sonicated; after 72 h, an aliquot of 0.6 ml was transferred into a 5-mm NMR sample tube. The resulting solution was at 2.5 mM concentration for both compounds. Reference baicalin solution was made by the same method without CD. In the beginning of our experiments, minimal precipitation was observed. Therefore, the concentration of baicalin in the final solution was reduced from 10.0 to 2.5 mM, in which concentration was still enough for the characterization of baicalin-cyclodextrin inclusion complexes and gave sufficient signal/noise ratio. Chemical shifts are given in ppm and are referenced to the residual solvent signal (DMSO-*d*_*6*_: δH = 2.50 ppm; δC = 39.50 ppm). For investigation of inclusion complexes, ^1^H spectrum was taken from 64 scans, 24 k data points, the sweep width was 6400 Hz. For the ROESY spinlock, a mixing time of 400 ms was used; the number of scans was 16 and 4 k time domain points and 256 increments were applied. For complete assignment, 32 scans, 32 k data points and 6400 Hz spectral width were used for ^1^H measurement and 16,384 scans, 64 k data points and 24,154 Hz sweep width were applied for ^13^C measurement. In case of the gHSQC spectrum, data points were acquired with 1 k × 512 and 4 scans were used. In the gHMBC experiment, 1 k × 256 data points and 8 scans were applied. gCOSY measurement was taken from 1 k × 256 data points and 8 scans. The processing was carried out by using a cosine-bell window function, single zero filling and automatic baseline correction.

### Molecular Modelling of the Binding into Cyclodextrin

All computations were carried out with the Gaussian09 program package (Gaussian Inc., USA, Wallingford CT, 2009) using convergence criteria of 3.0 × 10^−4^, 4.5 × 10^−4^, 1.2 × 10^−3^ and 1.8 × 10^−3^, for the gradients of the root mean square (RMS) force, maximum force, RMS displacement and maximum displacement vectors, respectively. The 3D structures of various cyclodextrin structures, like *β*-CD, *γ*-CD, *RAMEB*-CD and *SBE-β*-CD as well as their complexed forms with baicalin were optimized at B3LYP/6-31G(d) level of theory and compared to each other [27].

## RESULTS AND DISCUSSION

### Determination of the Dissociation Constants of Baicalin

In baicalin, there are 12 protons connected to carbon atoms, but in ^1^H NMR spectra, only the singlets of H8 and H3 on ring A and the doublet of the aromatic H2’,6′ protons can be easily assigned, while the other aromatic protons occur in complex spectrum of multiplets. The protons of the monosaccharide unit overlap with each other and with the signals of the antioxidant ascorbic acid. The suppression of the large water signal also interferes with the observation of certain NMR signals. Thus, during the NMR-pH titration, we followed the above-mentioned three chemical shift signal sets.

Since protonation processes are instantaneous on the NMR chemical shift time scale, the observed chemical shift (*δ*^obsd^) of a certain nucleus can be expressed as a weighted average of chemical shifts of the non-, mono-, di- and triprotonated forms of baicalin (B):

2 $$ {\delta}^{\mathrm{obsd}}={\delta}_{{\mathrm{B}}^{3\hbox{-} }}{x}_{{\mathrm{B}}^{3\hbox{-} }}+{\delta}_{{\mathrm{H}\mathrm{B}}^{2\hbox{-} }}{x}_{{\mathrm{H}\mathrm{B}}^{2\hbox{-} }}+{\delta}_{{\mathrm{H}}_2{\mathrm{B}}^{-}}{x}_{{\mathrm{H}}_2{\mathrm{B}}^{-}}+{\delta}_{{\mathrm{H}}_3\mathrm{B}}{x}_{{\mathrm{H}}_3\mathrm{B}} $$

where weighting factors are mole fractions that can be expressed in terms of stepwise protonation macroconstants and the actual hydrogen ion concentration. For example, ${x}_{{\mathrm{H}}_2{\mathrm{B}}^{-}}$ is:

3 $$ {x}_{{\mathrm{H}}_2{\mathrm{B}}^{-}}=\frac{K_1{K}_2{\left[{\mathrm{H}}^{+}\right]}^2}{1+{K}_1\left[{\mathrm{H}}^{+}\right]+{K}_1{K}_2{\left[{\mathrm{H}}^{+}\right]}^2+{K}_1{K}_2{K}_3{\left[{\mathrm{H}}^{+}\right]}^3} $$

Combining and rearranging Eqs. (2) and (3) yield Eq. (4) that can be directly fitted to the ^1^H-NMR titration curve of each nucleus observed.

4 $$ {\delta}^{\mathrm{obsd}}=\frac{\delta_{{\mathrm{B}}^{3\hbox{-} }}+{\delta}_{{\mathrm{H}\mathrm{B}}^{2\hbox{-} }}{K}_1\left[{\mathrm{H}}^{+}\right]+{\delta}_{{\mathrm{H}}_2{\mathrm{B}}^{-}}{K}_1{K}_2{\left[{\mathrm{H}}^{+}\right]}^2+{\delta}_{{\mathrm{H}}_3\mathrm{B}}{K}_1{K}_2{K}_3{\left[{\mathrm{H}}^{+}\right]}^3}{1+{K}_1\left[{\mathrm{H}}^{+}\right]+{K}_1{K}_2{\left[{\mathrm{H}}^{+}\right]}^2+{K}_1{K}_2{K}_3{\left[{\mathrm{H}}^{+}\right]}^3} $$

The logarithms of the obtained protonation constants correspond to the negative logarithms of dissociation constants, namely p*K*_a1_ = log*K*_3_, p*K*_a2_ = log*K*_2_ and p*K*_a3_ = log*K*_1_ [28].

**Fig. 1:**
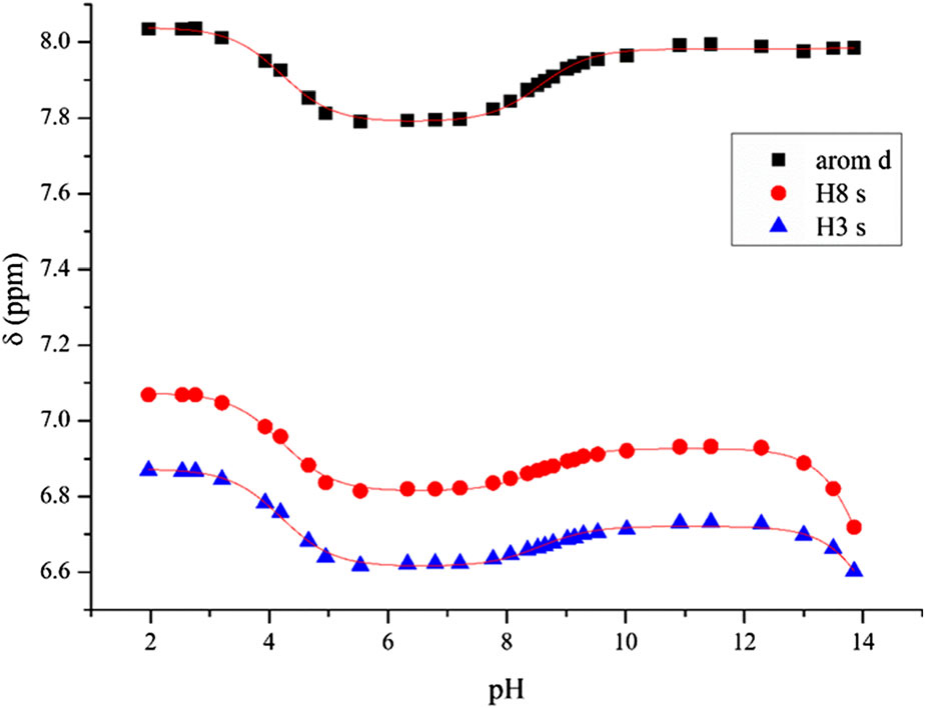
NMR-pH titration curves of the H2′,6′ (arom d) and H8 and H3 protons of baicalin. Computer fits of Eq. (4) are shown in solid lines

The NMR-pH titration curves can be seen in Fig. 1, while the obtained dissociation constants are summarized in Table I. The titration curves show that each observed nucleus displays chemical shift changes upon dissociation of each acidic proton.

**Table I:** Acid Dissociation Constants of Baicalin and Its Methyl Ester

	Baicalin		Baicalin methyl ester	
	AVG	SD	AVG	SD
p*K*_a1_	4.21	0.02	8.78	0.05
p*K*_a2_	8.56	0.03	> 14	–
p*K*_a3_	> 14	–	–	–

The methyl ester of baicalin has no free carboxyl functional group; therefore, two dissociation constants could be fitted only. The obtained p*K*_a_ values can be found in Table I.

The first acid dissociation constant (p*K*_a1_) obviously belongs to the carboxyl group of baicalin, because the methyl ester derivate (without a free carboxyl group) has no dissociation constant in the acidic region. Furthermore, two independent studies established that the UV spectrum of baicalin shows no profound absorption variation, just a slight increase in absorption intensity upon the increasing solubility of baicalin in less acidic solutions [10, 29]. The two phenolic hydroxyl groups on C5 and C6 on ring A would cause a marked bathochromic shift upon their deprotonation [30].

Thus, p*K*_a2_ of baicalin and the analogous p*K*_a1_ of baicalin methyl ester belong to one kind of the phenolic hydroxyl groups. The second phenolic hydroxyl deprotonation takes place above pH 14, but the exact value of the p*K*_a3_ of baicalin (or the analogous p*K*_a2_ of its methyl ester) cannot be determined with sufficient certainty. Due to the reasons below:

- the pH value of highly alkaline solutions can only be determined with indicator molecules, like 1-methylguanidine, but their chemical shift is also influenced by the changing ionic strength and solvation at these extreme pH values [31].
- the chemical shift of baicalin is also influenced by the changing ionic strength and solvation above pH 13, apart from the concurrent deprotonation.
- the rapid oxidation of baicalin above pH 13, even in the presence of a large excess of ascorbic acid, makes the observation of its signals very difficult and uncertain.

The high p*K*_a3_ values (above pH 13) of other biologically active catechols, like dopamine and epinephrine, can be determined only with uncertainty for similar reasons [32].

A UV-pH titration attempted to determine the p*K*_a2_ and p*K*_a3_ values of baicalin and claimed the 7.6 and 10.1, respectively [10]. However, these results are highly dubious in light of the oxidizability of baicalin in alkaline solutions. Namely, the change in the UV-spectra of baicalin is not only the result of protonation, but also that of the decomposition. With the help of ascorbic acid, we could prevent this oxidative process, and during NMR study, we could also monitor the pH range where the molecule has remained intact. The stability issues of baicalin were evaluated in buffered aqueous solutions at different pH values (2.0, 3.0, 4.5, 6.8, 7.4 and 9.0) and temperatures (4, 25 and 40°C). Acidic environment and low temperature were protective factors to preserve the integrity of baicalin [33].

**Fig. 2:**
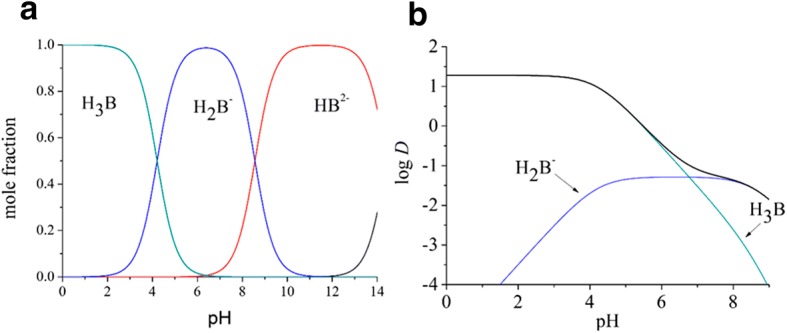
The species distribution diagram (**a**) and the lipophilicity profile of baicalin (**b**)

The knowledge of p*K*_a_ values allows the calculation of species distribution diagram of baicalin (Fig. 2a), showing the mole fraction of its variously charged forms as a function of pH.

The neutral form of baicalin (H_3_B) is dominant up to pH 4.21. The monoanionic form (H_2_B^−^) reaches its maximum at the pH of blood (pH 7.40), with 93.5%. The dianionic form (HB^2−^) becomes the dominant one above pH 8.56, while the trianionic form (B^3−^) starts to appear only in extremely alkaline solutions that have little relevance to biochemical or physiological processes.

### Determination of the Partition Coefficients of Baicalin

When more than one electrical species is present in solution, the observed ratio of concentrations in partition experiments is the distribution coefficient (*D*), which takes into account the intrinsic species-specific lipophilicity of the various electrical species present (*P*_i_), and their mole fractions in the aqueous phase (*x*_i_).

For baicalin, the pH-dependent distribution coefficient is the sum of four products:

5 $$ {D}_{\left(\mathrm{pH}\right)}=\Sigma {x}_{\mathrm{i}}{P}_{\mathrm{i}}={x}_{{\mathrm{B}}^{3\hbox{-} }}{P}_{{\mathrm{B}}^{3\hbox{-} }}+{x}_{{\mathrm{H}\mathrm{B}}^{2\hbox{-} }}{P}_{{\mathrm{H}\mathrm{B}}^{2\hbox{-} }}+{x}_{{\mathrm{H}}_2{\mathrm{B}}^{-}}{P}_{{\mathrm{H}}_2{\mathrm{B}}^{-}}+{x}_{{\mathrm{H}}_3\mathrm{B}}{P}_{{\mathrm{H}}_3\mathrm{B}} $$

where *x*_i_ mole fractions are the pH-dependent quantities, while *P*_i_ parameters are the pH-independent ones. The lipophilicity profile (the variation of log *D* as a function of the aqueous pH) of a drug is essential in understanding its pharmacokinetic behaviour [34].

The log *D* value of 1.28 (0.08) measured with a standardized 0.15 M HCl solution characterizes the log *P* value of the neutral form. In such acidic solutions, it is the dominant species and exhibits obviously higher lipophilicity than the anionic forms. In a previous study, the log *P* of baicalin was reported to be “1.27 (pH 7)”. It is not exactly clear what the authors meant by this, probably they calculated the log *P* of baicalin from a log *D* measured at pH 7 [10].

However, our log *D* value of − 1.22 (0.02) measured with a pH 7.40 phosphate buffer is a composite one, where contributions of both the neutral and the monoanionic forms are important and comparable. The relative concentration of the dianionic form is only 6.5% here, and due to its obviously smaller partition coefficient, it can be neglected at this physiological pH. Thus, Eq. (6) can be simplified as

6 $$ {D}_{\left(\mathrm{pH}\ 7.40\right)}={x}_{{\mathrm{H}}_2{\mathrm{B}}^{-}}{P}_{{\mathrm{H}}_2{\mathrm{B}}^{-}}+{x}_{{\mathrm{H}}_3\mathrm{B}}{P}_{{\mathrm{H}}_3\mathrm{B}} $$

The mole fraction data can be calculated similarly as shown in Eq. (3); thus, the partition coefficient of the anionic form can be obtained. The log *P* value of the anion turns out to be − 1.28; thus, there is a 2.56 log unit difference between the lipophilicity of the neutral and the anionic form. For phenols, the typical difference in the octanol/water system is around 3 log units, further verifying our results [34]. These partition coefficients allow the construction of the lipophilicity profile of baicalin, which can be seen in Fig. 2b. The broad black line is the overall lipophilicity profile of the molecule, the sum of the contributions of its two important species. The distribution coefficient becomes monotonically smaller as the pH is increased. The contribution of the neutral form is dominant up to pH 6.77, but at higher pH values, the anionic form is the more important one for the distribution coefficient.

### Determination of Thermodynamic Solubility by Saturation Shake-Flask Method

Baicalin is a weak triprotic acid (p*K*_a1_: 4.21, p*K*_a2_: 8.56, p*K*_a3_: >14) with remarkable pH-dependent solubility. The water solubility of baicalin was determined in distilled water (67.03 ± 1.60 μg/ml), which is in close agreement with literature data [6]. The difference could be explained by dissimilar sample preparation methods, in particular, equilibration time. In the highly acidic environment of stomach, the neutral form of baicalin is overwhelmingly dominant, so the measured solubility can be considered as the intrinsic solubility of baicalin (11.64 ± 0.44 μg/ml) at pH 1.2 SGF. As the pH increases, the ionization processes contribute significant solubility improvement (10,504 ± 330 μg/ml) at pH 6.8 SIF, where baicalin exists decisively in monoanionic form ([H_2_B]^−^: 98.0%). Significant solubilizing impact of FaSSGF (33.21 ± 0.72 μg/ml) was found to be correlated to compendial pH 1.2 SGF. The ~ 3 times increase in solubility can be explained by the presence of surface active agents (lecithin, taurocholate) in FaSSGF. Surprisingly, FaSSIF did not live up to our expectations. Instead of a remarkable solubilizing effect, a minor decrease (8111 ± 472 μg/ml) of thermodynamic solubility was found correlated to compendial pH 6.8 SIF (Table II). Similar results were observed in the case of weakly acidic furosemide and niflumic acid by Takács-Novák et al. [11]. The solubility of acidic zafirlukast was also negatively influenced by interactions of bile salt and soy lecithin, whereas the amphiphilic molecules exhibited significant positive effect for the weak base carvedilol [35]. The phenomena could be explained by the fact that taurocholate and lecithin micelles possess net negative charge; thus, an electrostatic repulsion exists between the solute anion and the micelle. In the case of neutral and cationic APIs, repulsive forces can be neglected.

**Table II:** Thermodynamic Solubility (μg/ml) of Baicalin in Various Compendial and Biorelevant Media

	AVG	SD
*Aqua dest.*	67.03	1.60
*pH = 1.2 (SGF)*	11.64	0.44
*pH = 6.8 (SIF)*	10,504	330
*pH = 1.6 (FaSSGF)*	33.21	0.72
*pH = 6.5 (FaSSIF)*	8111	472

### Phase Solubility Study

Phase solubility analysis can provide valuable information about changes in drug solubility when it interacts with different concentrations of CDs. Since the changes of physicochemical and biological properties of a drug are dependent on the stability constant of CD complexes, it is essential to determine this parameter accurately [36].

**Fig. 3:**
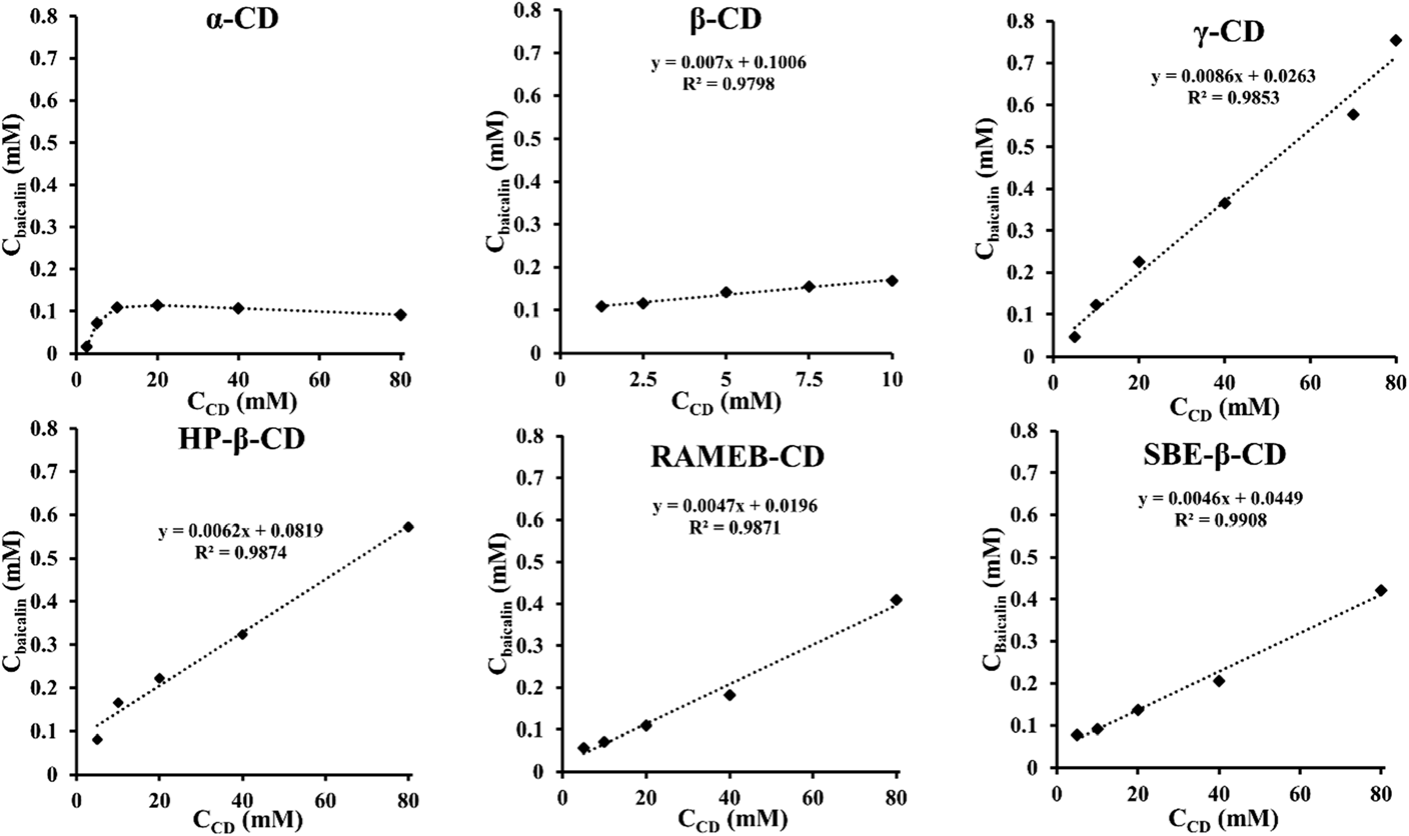
Phase solubility profiles of baicalin and various CDs

The phase solubility profile of baicalin-α-cyclodextrin complex showed a typical B_S_-type solubility curve, where the initial ascending portion is followed by a plateau region and then a slight decrease in total baicalin solubility accompanied by precipitation of complex. Baicalin-*β-*, *γ-*, *HP-β-*, *RAMEB-*, *SBE-β-*CD complexes revealed a linear enhancement in solubility of baicalin upon addition of increasing amounts of CD (Fig. 3). This indicates that the complexation belongs to the A_L_-type, assuming 1:1 binding stoichiometry. Solubility enhancement of 5.47 times was demonstrated by γ-CD encapsulation (67.03 μg/ml *vs.* 366.64 μg/ml) compared to solubility of baicalin in DW. In case of RAMEB, SBE-β-CD and HP-β-CD, the solubility improvement was significant, but less expressed, 2.88, 2.55 and 1.59 times, respectively. The inclusion complex of α- and β-CD did not reveal significant solubility enhancement. Based on Eq. (1), the apparent stability constants (*K*_*1:1*_) of host-guest complexes were calculated (Table III).

**Table III:** Apparent Stability Constants (K_1:1_) and Types of Phase Solubility Diagrams of Baicalin-CD Systems at 25°C

	*K*_*1:1*_	Type
*Α*	–	B_S_
*Β*	70.1	A_L_
*Γ*	329.8	A_L_
*HP-β*	76.2	A_L_
*RAMEB*	240.9	A_L_
*SBE-β*	102.9	A_L_

The largest *K*_*1:1*_ was found in case of *γ*-CD (329.8 M^−1^), which can well be explained by the fact that *γ*-CD is neutral and has wide internal cavity (8 glucopyranoside units). The order of stability constants was as follows: *γ*-CD > *RAMEB*-CD > *SBE-β*-CD > *HP-β-*CD > *β-*CD. The three most promising CDs were selected and examined further (*see sections* “Signal Assignment of Baicalin. Characterization of Baicalin-Cyclodextrin Inclusion Complexes” and “Molecular Modelling of the Baicalin-Cyclodextrin Complexations”).

### Signal Assignment of Baicalin. Characterization of Baicalin-Cyclodextrin Inclusion Complexes

The NMR analysis of baicalin was carried out in DMSO-*d*_*6*_ for complete assignment and published data were completed and corrected [37, 38]. The ^1^H NMR chemical shifts are in δ ppm (DMSO-*d*_*6*_, 400 MHz): 13.50–12.12 (1H, brs, COOH); 12.60 (1H, brs, C-5-OH); 8.70 (1H, brs, C-6-OH); 8.07 (2H, dm, *J =* 7.4 Hz, H-2′,6′); 7.67–7.54 (3H, m, H-3′,4′,5′); 7.05 (1H, s, H-8); 7.02 (1H, s, H-3); 6.05–4.65 (1H, brs, C-4”-OH); 5.53 (1H, d, *J =* 3.9 Hz, C-2″-OH); 5.34 (1H, d, *J =* 3.7 Hz, C-3″-OH); 5.25 (1H, d, *J =* 7.4 Hz, H-1″); 4.08 (1H, d, *J =* 9.5 Hz, H-5″); 3.52–3.26 (3H; m; H-2″,3″,4″). ^13^C NMR data in δ ppm (DMSO-*d*_*6*_, 100 MHz): 182.6 (C-4); 170.1 (C-6″); 163.6 (C-2); 151.3 (C-7); 149.2 (C-9); 146.8 (C-5); 132.1 (C-4′); 130.9 (C-1′); 130.6 (C-6); 129.2 (C-3′,5′); 126.4 (C-2′,6′); 106.1 (C-10); 104.8 (C-3); 99.9 (C-1″); 93.7 (C-8); 75.5 (C-5″); 75.3 (C-3″); 72.8 (C-2″); 71.3 (C-4″).

**Fig. 4:**
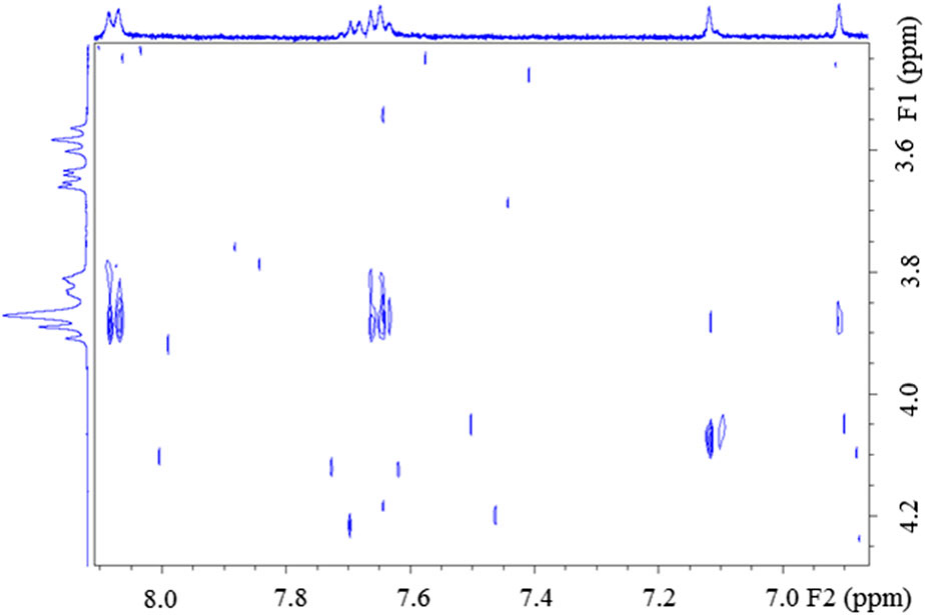
2D ROESY NMR spectrum of Baicalin-γ-CD complex; 400 MHz; 25°C

To obtain direct evidences for the interaction between baicalin and CDs, ROESY experiments were also carried out. In the case of *γ*-CD, unequivocal interaction was seen for H-3 protons of the CDs and aromatic protons (H-2′,4′ and H-3′,5′) of baicalin (Fig. 4). Weaker, but appreciable cross-peaks were shown also for H-5 protons of *γ*-CD with the aromatic protons (H-2′,4′ and H-3′,5′) and between H-3 of *γ*-CD and H-3 of baicalin. Interestingly, H-8 of baicalin had crosspeak only with H-5″. For *RAMEB-*CD, cross-peaks between the same moieties were found but at lower intensity. The baicalin-*SBE-β*-CD complex gave only the H-3 (baicalin)–H-3 (CD) interaction near to the noise.

**Fig. 5:**
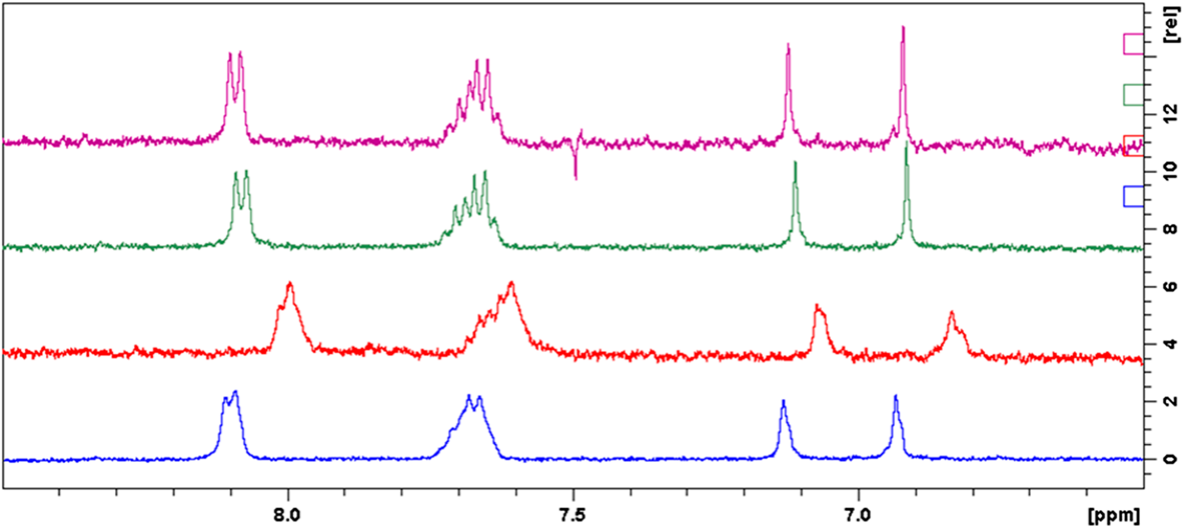
^1^H NMR spectra of baicalin (blue), baicalin-γ-CD (red), baicalin-RAMEB-CD (green) and baicalin-SBE-β-CD (purple); 400 MHz; 25°C

Proton chemical shifts in 1D ^1^H NMR spectra also differ before and after forming inclusion complexes (Table IV, Fig. 5.). All protons of the A, B and C rings were affected by the interaction with CDs. Most promoted chemical shift change was found for the *γ*-CD complex: *ca.* 0.1 ppm for H-3 and H-2′,6′; *ca.* 0.06 ppm for H-8 and H-3′,4′,5′. In case of *SBE-β* and *RAMEB*-CD inclusion complexes, the chemical shift changes were in the range of 0.01–0.02 ppm.

**Table IV:** ^1^H NMR Chemical Shifts of Baicalin in Its Solution and Inclusion Complexes; 400 MHz; 25°C

	Baicalin	*γ*-CD + Baicalin		*RAMEB-*CD + Baicalin		*SBE-β-*CD + Baicalin	
	δ (ppm)	δ (ppm)	Δδ (ppm)	δ (ppm)	Δδ (ppm)	δ (ppm)	Δδ (ppm)
H-3	6.9331	6.8348	− 0.0983	6.9145	− 0.0186	6.9211	− 0.0120
H-8	7.1301	7.0652	− 0.0649	7.1096	− 0.0205	7.1223	− 0.0078
H-3′,-4′,-5′	7.6811 7.76–7.60	7.6240 7.72–7.55	− 0.0571	7.67217.7434–7.6028	− 0.0090	7.6652 7.7365–7.5939	− 0.0159
H-2′,-6′	8.0986	8.0043	− 0.0943	8.0799	− 0.0187	8.0908	− 0.0078

Most clear evidences about complexation could be gained from the sample with *γ*-CD. ROESY and ^1^H NMR results suggest that H-2′,6′ and H-3 are inserted into the cavity, because these protons underwent the largest chemical shift change in ^1^H spectrum, and NOE interactions could also be developed with H-3 of CD. H-3′,4′,5′ show also NOE correlation with H-3 of CD, but a decreased chemical shift change could be detected, thus these protons are in the cavity, but near the rim of the CD and became not significantly shielded. Since aromatic protons have more intensive through-space interaction with H-3 of CD, than H-5 protons, it could be concluded, that the aromatic moiety is closer to the wide rim. Concerning the complexation with *RAMEB*-CD, analogous interactions could be observed as in case of solution with *γ*-CD, but weaker NOE interactions and moderate chemical shift changes could be observed. The results for baicalin-*SBE-β*-CD suggest only weak interaction between baicalin and the CD.

### Molecular Modelling of the Baicalin-Cyclodextrin Complexations

**Fig. 6:**
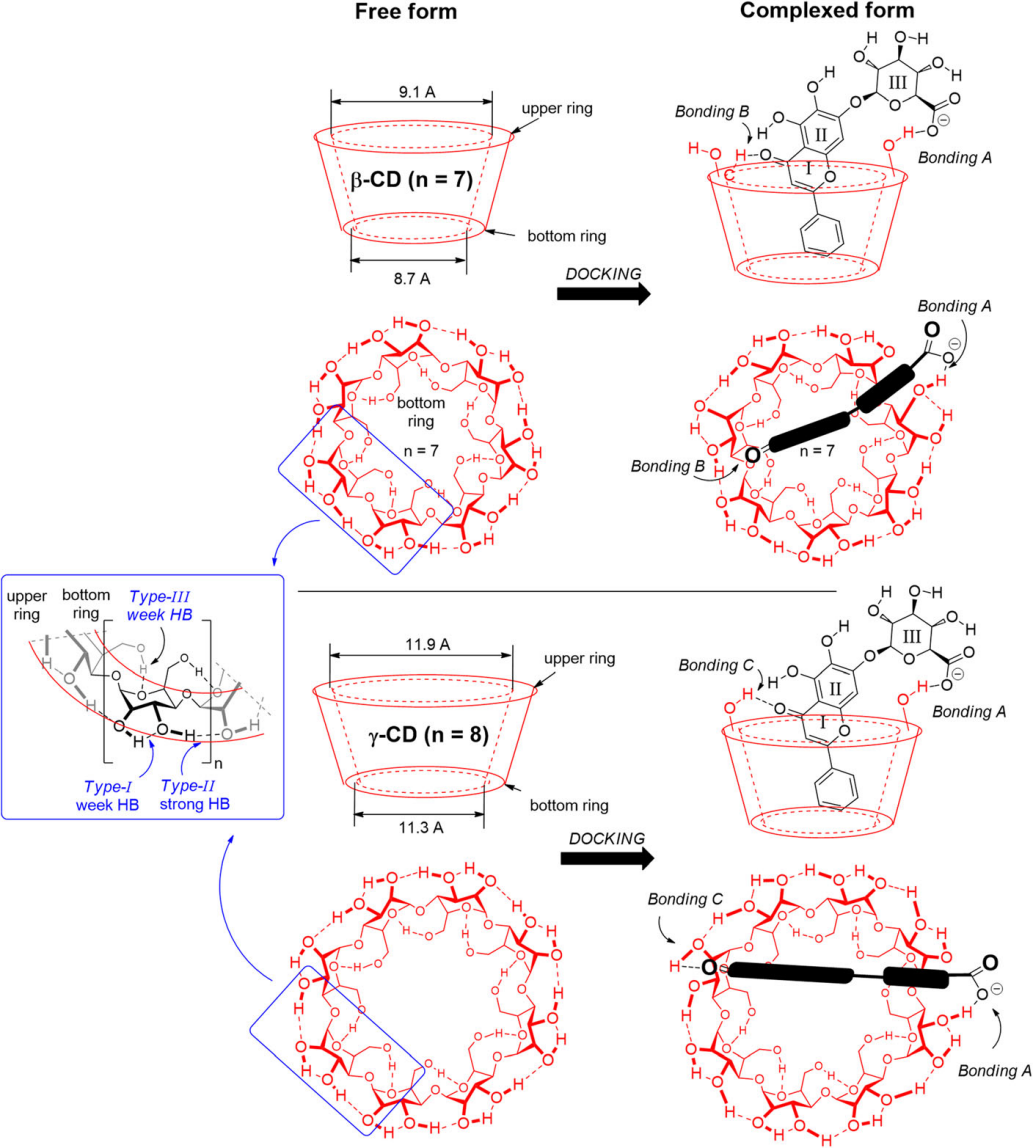
Different bonding types between baicalin and β-, γ-cyclodextrins (CD). For β-CD, the bonding pattern consists of Bonding-A and Bonding-B, while for γ-CD, it is Bonding-A and Bonding-C obtained by B3LYP/6-31G(d) level of theory

In the structures of CD, three different hydroxyl groups can be distinguished, two at the upper ring and one at the bottom ring for each hexose unit. According to computational study at B3LYP/6-31G(d) level of theory, anionic baicalin molecule docks and accommodates differently into the *β*-CD and *γ*-CD. In the most preferred arrangement of the baicalin in *β*-CD, it breaks one of the weak hydrogen bonds (HB) of *type I* OH at the upper ring and forms a strong HB (*Bonding-A*) with the carboxylate group of baicalin on *ring-III*, as illustrated in Fig. 6. The carbonyl group on the *ring-I* also forms a weak interaction with a neighbouring C–H of the opposite hexose.

The bonding pattern is different with *γ*-CD. Beside the same *Bonding-A*, the carbonyl group on the *ring-I* forms a much stronger HB with the *Type-II* OH group at the opposite site (*Bonding-C*). This difference can be revealed in their bonding energy values, listed in Table V. The calculated complexation energy in vacuum is − 162.4 kJ mol^−1^ for *β*-CD, while it is significantly stronger for *γ*-CD (− 181.5 kJ mol^−1^), exhibiting 19.1 kJ mol^−1^ beneficial energy difference in favour of *γ*-CD. This bonding pattern difference can be explained in terms of the diameter, related to the respective 7 and 8 glucose units in *β*- and *γ*-CD cyclodextrins. The strongest interaction is forming between the carboxylate and one of the OH groups of CD (*Bonding-A*), making a strong and rigid anchor. In the case of even number (*n* = 8; *γ*-CD), the opposite site provides beneficial OH group for the baicalin carbonyl (*Bonding-C*), while for odd number (*n* = 7; *β*-CD), the opposing wall can offer only a C–H group (*Bonding-B*).

**Table V:** Energy Differences of the Complexation Process of the Four Types of Cyclodextrins (β-CD, γ-CD, RAMEB-CD and SBE-β-CD) Obtained by B3LYP/6-31G(d) Level of Theory

	Complexation energy (Δ*E*; kJ mol^−1^)	ΔΔ*E*; kJ mol^−1^ relative to *β*-CD
*β-CD*	− 162.4	0.0
*γ-CD*	− 181.5	− 19.1
*RAMEB-CD*	− 164.2	− 1.8
*SBE-β-CD*	− 41.0	+ 121.4

**Fig. 7:**
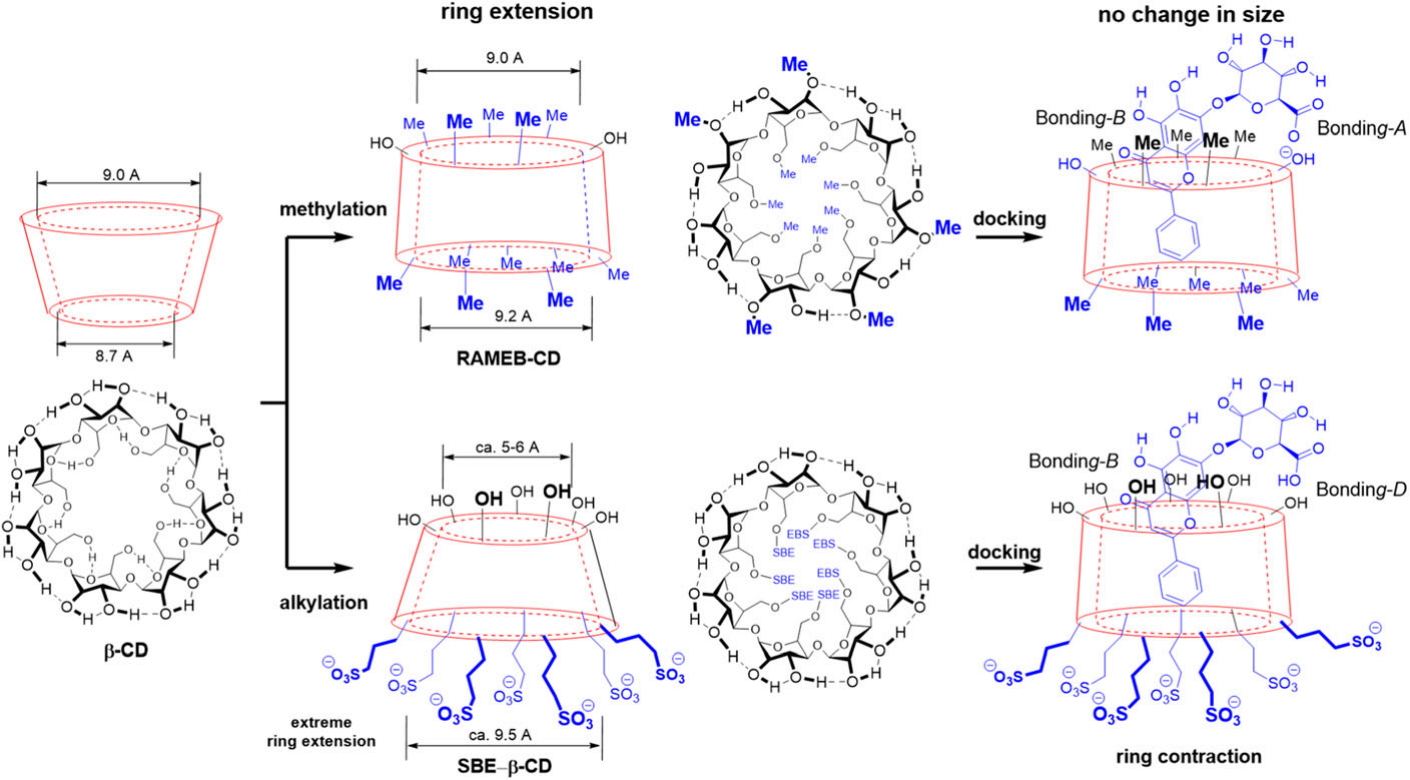
Shapes of the two modified β-cyclodextrins (RAMEB-CD and SBE-β-CD), obtained by B3LYP/6-31G(d) level of theory. On the right-hand side, structures represent the different bonding patterns between baicalin and modified β-CD. RAMEB-CD and SBE-β-CD consist of Bonding-A + Bonding-B and Bonding-B + Bonding-**D**, respectively

To model the partially methylated cyclodextrin, we constructed the model of *RAMEB*-CD according to literature data [39]. Here, the seven hydroxyl groups (OH; *type III*) at the bottom ring are completely methylated, while five out of the total of 14 the OH groups (*types I* and *II*) are methylated at the upper ring randomly. As Fig. 7 illustrates, the original cone shape of the *β*-CD has significantly changed by the partial methylation. Namely, the narrower bottom ring of the *β*-CD became extended and forms a loose ring structure. It makes significantly larger room to accommodate of the baicalin molecule. However, the *RAMEB*-CD still provides enough hydroxyl groups at the upper ring to form strong hydrogen bonding (*Bonding-A*), like the original *β*-CD, as illustrated in Fig. 6. The apolar bottom part of the CD can interact more comfortably with the phenyl substituent of the drug molecule. The computed complexation energy is little bit lower, than that for *β*-CD, which can support the experimental findings.

**Fig. 8:**
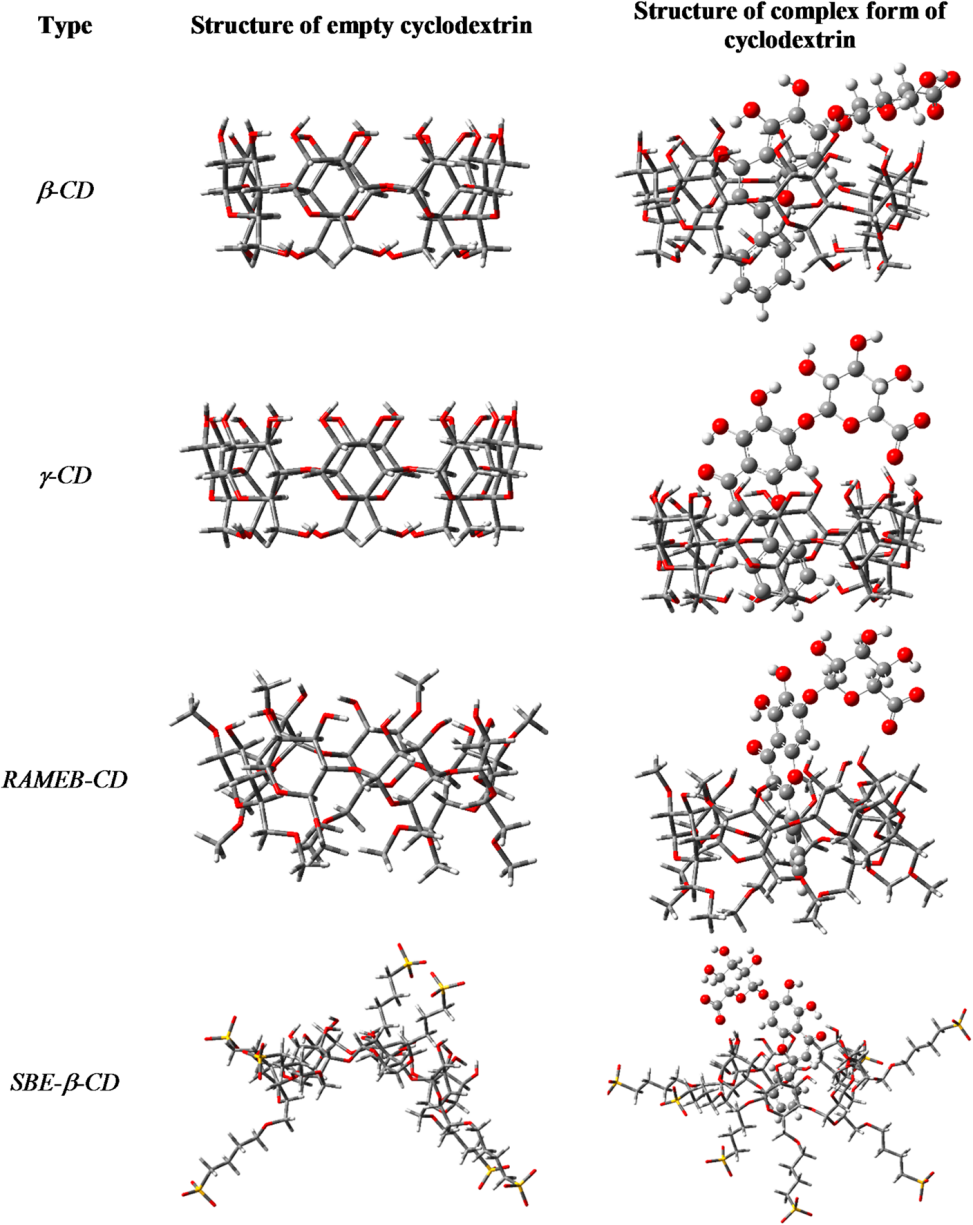
Optimized structures of various cyclodextrins and their complexed forms at B3LYP/6-31G(d) level of theory

The seven sulfobutylether groups in *SBE-*β*-*CD typically replace the seven *type III* OH groups. Supposing a complete deprotonation of the sulfonic acid, the seven anionic sulfonate groups expand the cyclodextrin ring extremely, due to the Coulomb repulsion of the anions. This effect opens the bottom end of the CD and closes the upper ring. During the complexation, the neutral form of baicalin needs to re-open the upper ring to get into the hole and forms a similar HB with one of the type I OH, but here the COOH points to the CD-OH (*Bonding-D*). The computed energy is far less beneficial, than that of for *β*-CD, which should not be realistic, compared to the experimental result. Here, the neglect of the whole solvent environment overestimates the coulomb repulsion, which lowers the level of reliability. The calculated complexation energies refer to vacuum, while in case of phase-solubility and NMR studies, a complex solvent system was utilized. The energy-minimised structures of CD complexes are demonstrated in Fig. 8.

## CONCLUSIONS

In this paper, the detailed physicochemical characterization of baicalin was demonstrated, which provided valuable information to design the proper formulation and delivery route for this BCS IV active phytopharmaceutical ingredient. Low bioavailability could be overcame by cyclodextrin complexation; therefore, six different derivates were examined to study solubility enhancement along with stability and geometry of inclusion complex. Molecular modelling analysis pointed out different binding patterns and complexation energy for each baicalin-CD complex. In accordance with our previous hypothesis, thermodynamically the most favourable complex proved to be baicalin-*γ*-CD host-guest complex (Δ*E* = − 181.5 kJ mol^−1^), in which fact was confirmed by phase-solubility (≈ 5.5 times solubility enhancement) and NMR spectroscopic measurements. The demonstrated theoretical and experimental methods could be adopted in the formulation development and analysis cyclodextrins containing neutral drugs. The future research direction is the solidification and optimization of baicalin-*γ*-CD inclusion complex and the phytopharmacological investigation in a Drug Delivery System.

## Abbreviatons

BCS: Biopharmaceutical Classification System
CD: Cyclodextrin
DDS: Drug Delivery System
FaSSGF: Fasted state simulated gastric fluid
FaSSIF: Fasted state simulated intestinal fluid
RAMEB-CD: Randomly methylated β-CD
SBE- *β*-CD: Sulfobutylether-*β*-CD
SGF: Simulated gastric fluid
SIF: Simulated intestinal fluid

## References

[1] Chen H, Gao Y, Wu J, Chen Y, Chen B, Hu J, Zhou J (2014). Exploring therapeutic potentials of baicalin and its aglycone baicalein for hematological malignancies. Cancer Lett.

[2] European Pharmacopoeia. 5.22 Names of herbal drugs used in traditional Chinese medicine. Scutellariae baicalensis radix 04/2011:2438. In: Ph Eur 9.0. 2018. EDQM Council of Europe, Strasbourg, France. p. 1262–1263.

[3] Li-weber M (2009). New therapeutic aspects of flavones : the anticancer properties of Scutellaria and its main active constituents Wogonin , Baicalein and Baicalin. Cancer Treat Rev.

[4] Shin SH, Bak S, Kim MK (2015). Baicalin , a flavonoid , affects the activity of human dermal papilla cells and promotes anagen induction in mice. Naunyn Schmiedeberg's Arch Pharmacol.

[5] Zhao Q, Cathie XC (2016). Scutellaria baicalensis , the golden herb from the garden of Chinese medicinal plants. Sci Bull.

[6] Wu H, Long X, Yuan F, Chen L, Pan S, Liu Y, Stowell Y, Li X (2014). Combined use of phospholipid complexes and self-emulsifying microemulsions for improving the oral absorption of a BCS class IV compound, baicalin. Acta Pharm Sin B.

[7] Xing J, Chen X, Zhong D (2005). Absorption and enterohepatic circulation of baicalin in rats. Life Sci.

[8] Dewey H. Barich, Mark T. Zell E. J. M.. Physicochemical properties, formulation and drug delivery. In: Drug Delivery: Principles and Applications. 2016. John Wiley & Sons, Inc., Hoboken, New Jersey. p. 35–48.

[9] Kalapos-Kovács B, Magda B, Jani M, Fekete Z, Szabó PT, Antal I, Krajcsi P, Klebovich I (2015). Multiple ABC Transporters Efflux Baicalin. Phytother Res.

[10] Liang RAN, Rui-Min HAN, Li-Min FU, Xi-Cheng AI, Zhang JP, Skibsted LH (2009). Baicalin in radical scavenging and its synergistic effect with β-carotene in antilipoxidation. J Agric Food Chem.

[11] Takacs-Novak K, Szoke V, Volgyi G, Horvath P, Ambrus R, Szabo-Revesz P (2013). Biorelevant solubility of poorly soluble drugs: rivaroxaban, furosemide, papaverine and niflumic acid. J Pharm Biomed Anal.

[12] Bloomer JC, Ambery C, Miller BE, Connolly P, Garden H, Henley N, Hodnett N, Keel S, Kreindler JL, Lloyd RS, Matthews W, Yonchuk J, Lazaar AL (2017). Identification and characterisation of a salt form of Danirixin with reduced pharmacokinetic variability in patient populations. Eur J Pharm Biopharm.

[13] Andreas CJ, Tomaszewska I, Muenster U, van der Mey D, Mueck W, Dressman JB (2016). Can dosage form-dependent food effects be predicted using biorelevant dissolution tests? Case example extended release nifedipine. Eur J Pharm Biopharm.

[14] Marques MRC, Loebenberg R, Almukainzi M (2011). Simulated biological fluids with possible application in dissolution testing. Dissolut Technol.

[15] Jakab G, Fülöp V, Bozó T, Balogh E, Kellermayer M, Antal I (2018). Optimization of Quality Attributes and Atomic Force Microscopy Imaging of Reconstituted Nanodroplets in Baicalin Loaded Self-Nanoemulsifying Formulations. Pharmaceutics.

[16] Li Ying, He Zhen-Dan, Zheng Qian-En, Hu Chengshen, Lai Wing-Fu (2018). Hydroxypropyl-β-cyclodextrin for Delivery of Baicalin via Inclusion Complexation by Supercritical Fluid Encapsulation. Molecules.

[17] Yue P, Xiao M, Xie Y, Ma Y, Guan Y, Wu Z, Hu PY, Wang YQ (2016). The roles of Vitrification of stabilizers/matrix formers for the Redispersibility of drug nanocrystals after solidification: a case study. AAPS PharmSciTech.

[18] Conceição Jaime, Adeoye Oluwatomide, Cabral-Marques Helena Maria, Sousa Lobo José Manuel (2018). Hydroxypropyl-β-Cyclodextrin and β-Cyclodextrin as Tablet Fillers for Direct Compression. AAPS PharmSciTech.

[19] Li J, Jiang Q, Deng P, Chen Q, Yu M, Shang J, Li W (2017). The formation of a host-guest inclusion complex system between β-cyclodextrin and baicalin and its dissolution characteristics. J Pharm Pharmacol.

[20] Loftsson T, Vogensen SB, Brewster ME (2007). Effects of Cyclodextrins on Drug Delivery Through Biological Membranes. J Pharm Sci.

[21] Coisne Caroline, Tilloy Sébastien, Monflier Eric, Wils Daniel, Fenart Laurence, Gosselet Fabien (2016). Cyclodextrins as Emerging Therapeutic Tools in the Treatment of Cholesterol-Associated Vascular and Neurodegenerative Diseases. Molecules.

[22] Klein S (2010). The use of biorelevant dissolution media to forecast the in vivo performance of a drug. AAPS J.

[23] Baka E, Comer JEA, Tak K (2008). Study of equilibrium solubility measurement by saturation shake-flask method using hydrochlorothiazide as model compound. J Pharm Biomed Anal.

[24] Orgovan G, Noszal B (2011). Electrodeless, accurate pH determination in highly basic media using a new set of (1)H NMR pH indicators. J Pharm Biomed Anal.

[25] Mazak K, Hosztafi S, Kraszni M, Noszal B (2017). Physico-chemical profiling of semisynthetic opioids. J Pharm Biomed Anal.

[26] Higuchi T, Connors KA (1965). Phase solubility studies. Adv Anal Chem Instrum.

[27] Becke AD (1993). Density-functional thermochemistry. III. The role of exact exchange. J Chem Phys.

[28] Mazak K, Noszal B (2016). Advances in microspeciation of drugs and biomolecules: species-specific concentrations, acid-base properties and related parameters. J Pharm Biomed Anal.

[29] Zhou Y, Yang Z-Y, Tang R-C (2016). Bioactive and UV protective silk materials containing baicalin - the multifunctional plant extract from Scutellaria baicalensis Georgi. Mater Sci Eng C Mater Biol Appl.

[30] Mazak K, Doczy V, Kokosi J, Noszal B (2009). Proton speciation and microspeciation of serotonin and 5-hydroxytryptophan. Chem Biodivers.

[31] Szakacs Z, Beni S, Noszal B (2008). Resolution of carboxylate protonation microequilibria of NTA, EDTA and related complexones. Talanta.

[32] Kiss T, Sovago I, Martin RB (1989). Complexes of 3,4-dihydroxyphenyl derivatives. 9. Aluminum(3+) binding to catecholamines and tiron. J Am Chem Soc.

[33] Feng Z, Zhou J, Shang X, Kuang G, Han J, Lu L, Zhang L (2017). Comparative research on stability of baicalin and baicalein administrated in monomer and total flavonoid fraction form of *Radix scutellariae* in biological fluids *in vitro*. Pharm Biol.

[34] Mazak K, Noszal B (2014). Drug delivery: a process governed by species-specific lipophilicities. Eur J Pharm Sci.

[35] Zhou Z, Dunn C, Khadra I, Wilson CG, Halbert GW (2017). Influence of physiological gastrointestinal surfactant ratio on the equilibrium solubility of BCS class II drugs investigated using a four component mixture design. Mol Pharm.

[36] Shelley H, Grant M, Smith FT, Abarca EM, Jayachandra BR (2018). Improved ocular delivery of Nepafenac by Cyclodextrin complexation. AAPS PharmSciTech.

[37] Wu S, Sun A, Liu R (2005). Separation and purification of baicalin and wogonoside from the Chinese medicinal plant Scutellaria baicalensis Georgi by high-speed counter-current chromatography. J Chromatogr A.

[38] Yuan Yuan, Hou Wenli, Tang Minhai, Luo Houding, Chen Li-Juan, Guan Y. Hugh, Sutherland Ian A. (2008). Separation of Flavonoids from the Leaves of Oroxylum indicum by HSCCC. Chromatographia.

[39] Przybylski C, Bonnet V, Cézard C (2015). Probing the common alkali metal affinity of native and variously methylated b-cyclodextrins by combining electrospray-tandem mass spectrometry and molecular modeling. Phys Chem Chem Phys.

